# Exploring TCGA database for identification of potential prognostic genes in stomach adenocarcinoma

**DOI:** 10.1186/s12935-020-01351-3

**Published:** 2020-06-23

**Authors:** Lin Zhou, Wei Huang, He-Fen Yu, Ya-Juan Feng, Xu Teng

**Affiliations:** 1 School of Information Science and Technology, University of Science and Technology of China, Hefei, 230026 Anhui China; 2 Beijing Key Laboratory for Tumor Invasion and Metastasis, Department of Biochemistry and Molecular Biology, School of Basic Medical Sciences, Capital Medical University, Beijing, 100069 China

**Keywords:** Tumour microenvironment, Stomach adenocarcinoma, TCGA, ESTIMATE algorithm, Prognosis

## Abstract

**Background:**

Stomach adenocarcinoma (STAD) is the fifth most prevalent cancer in the world and ranks third among cancer-related deaths worldwide. The tumour microenvironment (TME) plays an important role in tumorigenesis, development, and metastasis. Hence, we calculated the immune and stromal scores to find the potential prognosis-related genes in STAD using bioinformatics analysis.

**Methods:**

The ESTIMATE algorithm was used to calculate the immune/stromal scores of the STAD samples. Functional enrichment analysis, protein–protein interaction (PPI) network analysis, and overall survival analysis were then performed on differential genes. And we validated these genes using data from the Gene Expression Omnibus database. Finally, we used the Human Protein Atlas (HPA) databases to verify these genes at the protein levels by IHC.

**Results:**

Data analysis revealed correlation between stromal/immune scores and the TNM staging system. The top 10 core genes extracted from the PPI network, and primarily involved in immune responses, extracellular matrix, and cell adhesion. There are 31 genes have been validated with poor prognosis and 16 genes were upregulated in tumour tissues compared with normal tissues at the protein level.

**Conclusions:**

In summary, we identified genes associated with the tumour microenvironment with prognostic implications in STAD, which may become potential therapeutic markers leading to better clinical outcomes.

## Background

Stomach adenocarcinoma (STAD) is ranked as the fifth-most commonly diagnosed cancer and the third leading cause of cancer-related deaths worldwide as per the statistics of GLOBOCAN 2018 [1], with an estimated 679,100 new cases and 498,000 deaths occurring in China in 2015 [2]. Around 90–95% of all stomach cancers are adenocarcinoma. They are subdivided into cardia and non-cardia gastric cancers, respectively, based on whether the tumour is located near the gastro-oesophageal junction (cardia) or away from it [3]. Worldwide, the incidence rate of STAD is the highest in Asia, and among the Asian countries, China has the maximum incidence rate of STAD, accounting for 49.9% of global STAD cases [4]. In European countries, the 5-year survival rate varies from ~ 10 to 30% [5], and in China, from 30.2 to 35.9% [6]. To better understand the impact on tumour genetic composition of clinical outcomes, genome-wide gene expression repertoires, such as The Cancer Genome Atlas (TCGA) have been established to explore and discover large cohorts around the world [7]. Although extensive research has been conducted on the mechanism of the occurrence and development of STAD, the aetiology and pathogenesis of STAD still remain to be elucidated [8]. Hence, considering the high morbidity and mortality of STAD, it is essential to explore molecular markers that have a prognostic value of influencing the immune response from STAD patients.

The cells within the tumour microenvironment (TME) are an important component of the tumour tissue. An increasing number of evidences has elucidated the clinic pathological significance of TME in the prediction of treatment effects [9, 10]. The TME is the cellular milieu where the tumour is located. It consists of immune cells, mesenchymal cells, endothelial cells, along with inflammatory mediators and extracellular matrix (ECM) molecules [11, 12]. The cells and molecules in the TME are in a dynamic process, reflecting the evolutionary nature of cancer and jointly promoting immune escape, growth, and metastasis of tumours [13, 14]. Immune cells and stromal cells are the two main types of non-tumour components and considered to be of great value in the diagnosis and prognosis of tumours [7]. Therefore, understanding the molecular composition and function of TME are essential for the effective management of cancer progression and immune response in STAD. With the advent of the era of big data biology, bioinformatics analysis of large amounts of data has been made possible through a combination of biology, computer science, and information technology [15]. Its rapid development provides researchers with a more user-friendly and convenient platform to guide the implementation of basic experiments [16]. In 2013, Yoshihara et al. designed an algorithm called ESTIMATE to estimate stromal cells and immune cells in malignant tumour tissues with expression data. In this algorithm, the authors obtained immune and stromal scores to predict the TME by calculating the expression characteristics of specific molecular biomarkers in immune and stromal cells [17]. In addition, they used estimate scores to comprehensively evaluate the immune and stromal scores. In recent years, the ESTIMATE algorithm has been reported to be applied to glioblastoma [7], clear cell renal cell carcinoma [18], and colon cancer [19], thereby demonstrating the validity of this big-data based algorithm. However, there is no detailed analysis of the immune, stromal, and estimate scores of stomach adenocarcinoma.

In this study, the TME-related genes were obtained from the stomach adenocarcinoma datasets in TCGA database and the ESTIMATE algorithm was used to analyse the corresponding immune/stromal/estimate scores. Some core genes were obtained through the analysis of functional annotations and gene networks. Finally, the STAD dataset from the Gene Expression Omnibus (GEO) database was used to validate the acquired core genes, revealing their potential roles in the treatment of STAD.

## Methods

### Raw data

RNA-seq data for STAD patients were downloaded from TCGA database (https://tcga-data.nci.nih.gov/tcga/), the gene expression profile was measured experimentally using the Illumina HiSeq2000 RNA Sequencing platform by the University of North Carolina TCGA genome characterization centre. Clinical data such as age, TNM staging, gender, survival-time, and status were also downloaded from the cBioportal website (http://www.cbioportal.org/). We calculated the stromal/immune/estimate scores of the samples using the ESTIMATE algorithm (https://r-forge.r-project.org). The GSE84433 dataset from the GEO database was used for validation. The Human Protein Atlas (http://www.proteinatlas.org) was used to validate the immunohistochemistry of genes with prognostic values. Direct links to the immunohistochemistry images from the Human Protein Atlas are provided in the following:

**Table:** 

Gene	Normal	Tumor
BCHE	https://www.proteinatlas.org/ENSG00000114200-BCHE/tissue/stomach#img	https://www.proteinatlas.org/ENSG00000114200-BCHE/pathology/stomach+cancer#img
CNN1	https://www.proteinatlas.org/ENSG00000130176-CNN1/tissue/stomach#img	https://www.proteinatlas.org/ENSG00000130176-CNN1/pathology/stomach+cancer#img
CPED1	https://www.proteinatlas.org/ENSG00000106034-CPED1/tissue/stomach#img	https://www.proteinatlas.org/ENSG00000106034-CPED1/pathology/stomach+cancer#img
CYP1B1	https://www.proteinatlas.org/ENSG00000138061-CYP1B1/tissue/stomach#img	https://www.proteinatlas.org/ENSG00000138061-CYP1B1/pathology/stomach+cancer#img
SELP	https://www.proteinatlas.org/ENSG00000174175-SELP/tissue/stomach#img	https://www.proteinatlas.org/ENSG00000174175-SELP/pathology/stomach+cancer#img
VIP	https://www.proteinatlas.org/ENSG00000146469-VIP/tissue/stomach#img	https://www.proteinatlas.org/ENSG00000146469-VIP/pathology/stomach+cancer#img

### Differential expression analysis

Differential expression analysis was performed on the count matrix of the sample using the R package, DESeq2. The screening conditions for the differential genes were: Fold Change > | ±1.5|, adjusted p-values (padj) < 0.05. Heat maps of differential genes were drawn using the R-package, pheatmap.

### Enrichment analysis and PPI network

The Database for Annotation, Visualization and Integrated Discovery (DAVID) tool [20] was used to perform functional enrichment analysis of the differentially expressed genes (DEGs), the corresponding biological processes (BP), cell components (CC), and molecular functions (MF) were identified using Gene Ontology (GO) and the signalling pathways involved were identified using the Kyoto Encyclopedia of Genes and Genomes (KEGG). The protein–protein interaction (PPI) network was constructed using the Search Tool for the Retrieval of Interacting Genes (STRING) database [21], and the core genes were identified using the CytoHubba plug-in in Cytoscape software [22]. Module analysis for the detection of interaction networks was performed using the Molecular Complex Detection (MCODE) plug-in in the Cytoscape platform.

### Survival analysis

The survival curve is shown using the Kaplan–Meier curve, which is drawn using the R packages survival and survminer. This relationship was verified using a log-rank test. These analyses illustrate the relationship between differential genes and overall patient survival.

## Results

### Stromal and immune scores are associated with the TNM staging system and survival prognosis

**Fig. 1:**
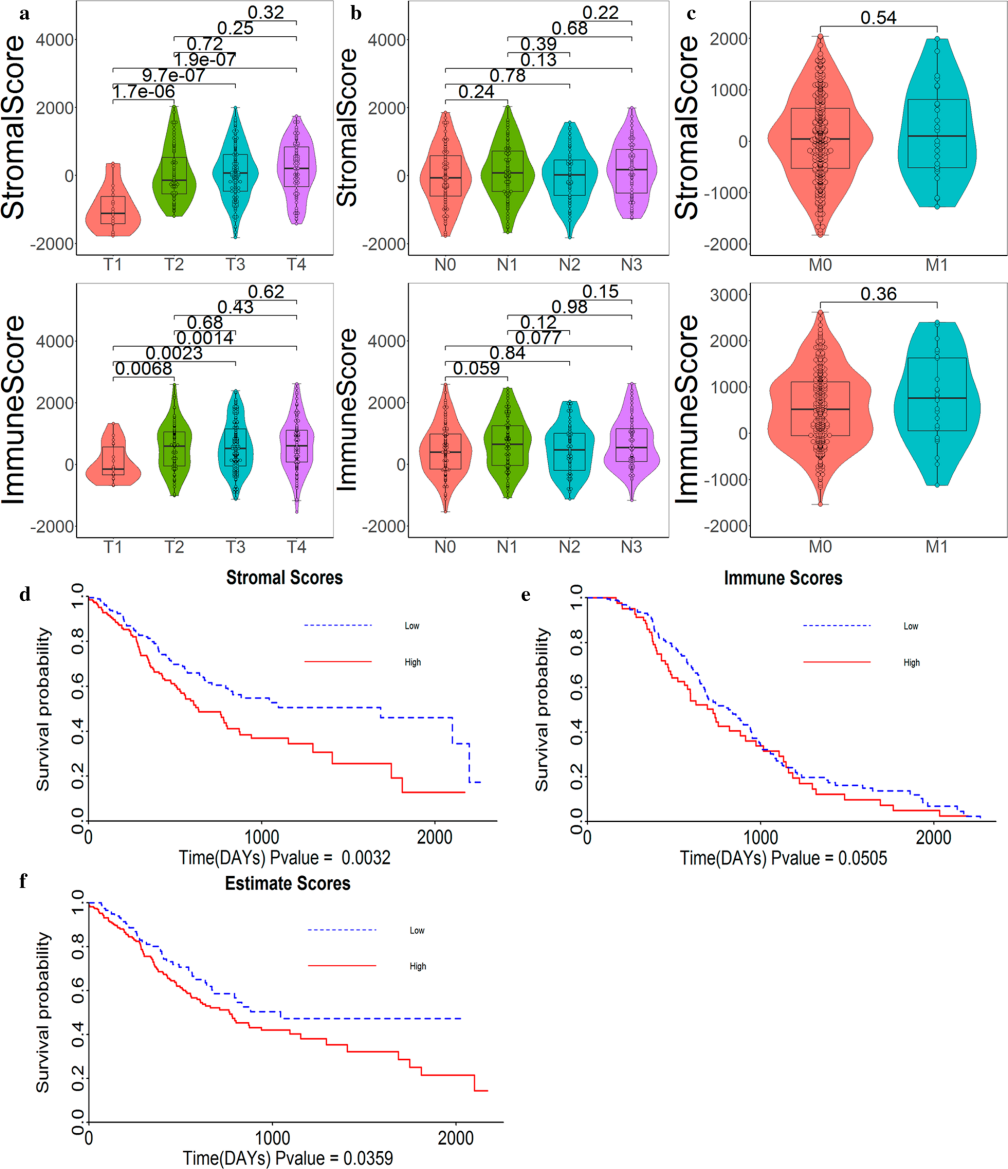
Stromal, immune, and estimate score distribution and survival analysis of STAD samples. **a** Score distribution of T staging. The violin plot shows a correlation between the T stage of STAD and the stromal score level. **b** Score distribution of N staging. The violin plot showed no significant association between the N stage and stromal/immune scores (p > 0.05). **c** Score distribution of M staging. The violin plot showed no significant association between the M stage and stromal/immune scores (p = 0.54 and p = 0.36, respectively). **d** From the survival curve, we can see that the high stromal score is related to the poor overall survival (p = 0.0032). **e** Similarly, high immune scores are associated with poor overall survival (p = 0.0505). **f** Estimate score is also related to overall survival (p = 0.0359)

We downloaded the RNA-seq gene expression matrix and clinical information data from 380 patients with STAD from TCGA database. Of all the samples, 63.9% were male and 36.1% were female, and 62.9% were white, 19.7% Asian, 14.2% were not reported, while others were black or African–American. The proportion of patients with T1–T2 and T3–T4 was 27.7% (n = 108) and 72.3% (n = 272), respectively. The proportion of patients with N0–N1 and N2–N3 was 58.4% (n = 221) and 41.6% (n = 159), respectively. Patients with M0 and M1 reached 92.7% (n = 343) and 7.3% (n = 37), respectively. The stromal scores and immune scores of all samples were obtained by ESTIMATE algorithm, and the score ranges were − 1832.01 to 2038.29 and − 1541.7 to 2619.69, respectively. From the aspect of tumour infiltration depth (T), the median stromal and immune scores in T1 stage are the lowest, and the order for stromal median score from T2 to T4 is: T2 < T3 < T4, order of immune scores is: T3 < T2 < T4, but the difference between them is not obvious (Fig. 1a). From the aspect of lymph node staging (N), the relationships between the median stromal and immune scores of the four stages were similar (without statistical significance) namely: N3 > N1 > N2 > N0, N1 > N3 > N2 > N0 (Fig. 1b). Finally, in terms of distant metastasis (M), the stromal and immune median scores are in the same order: M1 > M0, without statistical significance (Fig. 1c). From these data, it can be seen that there is an intense correlation between stromal/immune/estimate scores and the TNM staging system.

To analyse the potential relationship between the stromal/immune/estimate scores and the overall survival of the samples, we divided all samples into high and low score groups based on the positive/negative stromal/immune/estimate scores. The Kaplan–Meier survival curve showed that the high score group of the stromal scores has a lower survival rate than the low score group (Fig. 1d, p = 0.0032 in log-rank test). Similar phenomena were observed in the high and low score groups of the immune/estimate scores (Fig. 1e, p = 0.0505 in log-rank test, Fig. 1f, p = 0.0359 in log-rank).

### Differential expression and enrichment analysis of STAD cases based on stromal and immune scores

**Fig. 2:**
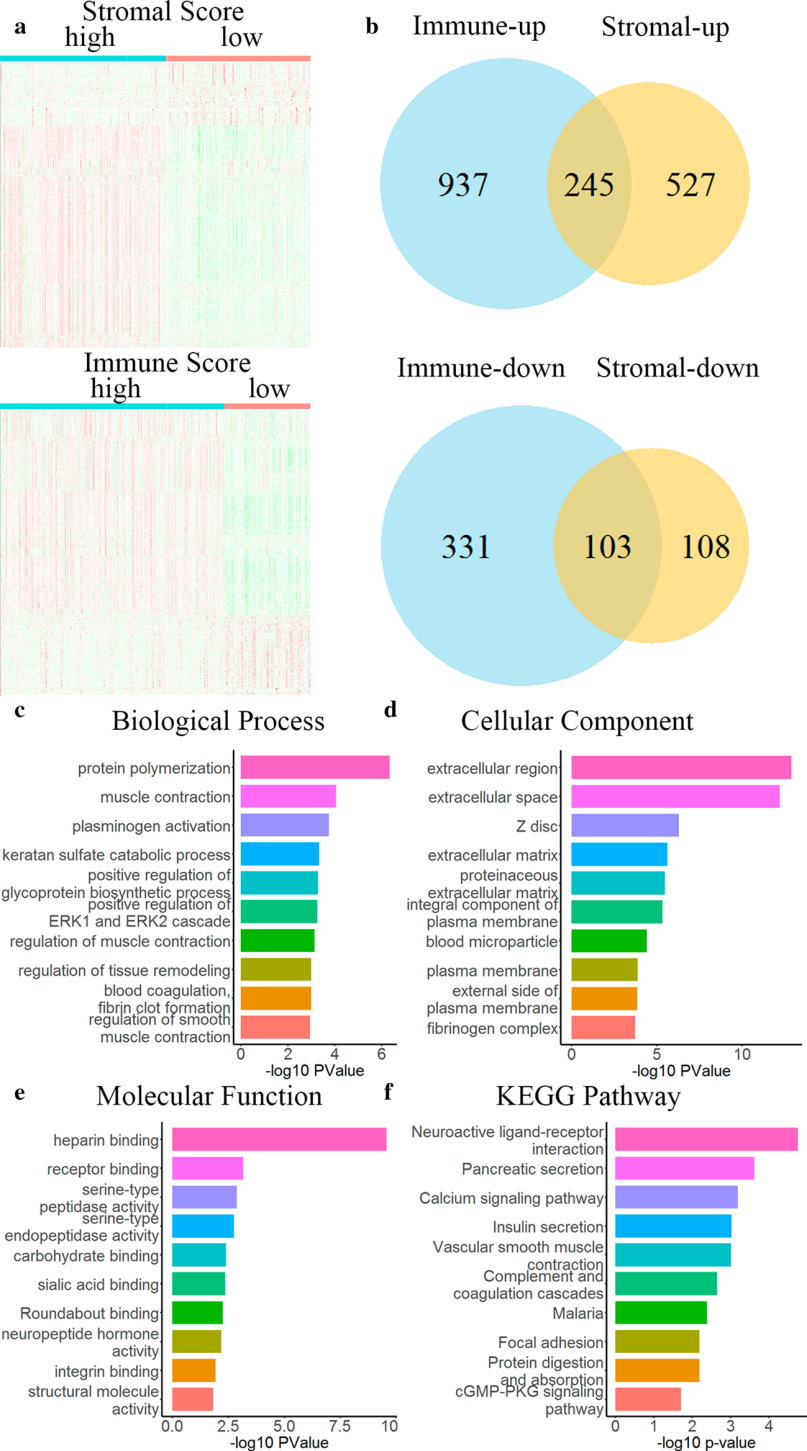
Differential and functional enrichment analysis of gene expression matrices in STAD samples. **a** Heat maps of the differential genes with stromal scores and immune scores of high score groups and low score groups (padj < 0.05, fold change > |±1.5|). **b** Venn diagram of consensus genes differentially expressed genes between the stromal and immune cell groups. **c**–**f** Top 10 GO terms from enrichment analysis and KEGG pathway analysis of up-regulated consensus genes

To reveal the relationship between the stromal and/or immune scores and the gene expression profile of the samples, we performed differential analysis of all RNA-seq data from 380 STAD cases in TCGA database. The heat map of the high/low scores of the stromal/immune scores revealed differential gene expression profiles between the samples, in which 772 up-regulated genes and 211 down-regulated genes (fold change > |± 1.5|, padj < 0.05) were obtained based on the difference in stromal scores, simultaneously, 1182 up-regulated genes and 434 down-regulated genes (fold change > |± 1.5|, padj < 0.05) were obtained based on the differential analysis of immune scores (Fig. 2a). As can be seen from the Venn diagram (Fig. 2b), there are 245 identical up-regulated genes and 103 identical down-regulated genes (Additional file 1: Table S1).

We performed functional enrichment analysis on the obtained 348 differential genes (245 up-regulated genes and 103 down-regulated genes), including GO: BP, GO: CC, GO: MF, and KEGG pathway analysis. Sorting by − Log10 (p-value), we list the top 10 terms of each section. GO functions are mainly enriched in inflammatory and immune responses, extracellular matrices, and heparin binding (Fig. 2c–e), while KEGG pathways are mainly enriched in neuroactive ligand-receptor interaction and insulin secretion (Fig. 2f).

### Survival analysis of differential genes

**Fig. 3:**
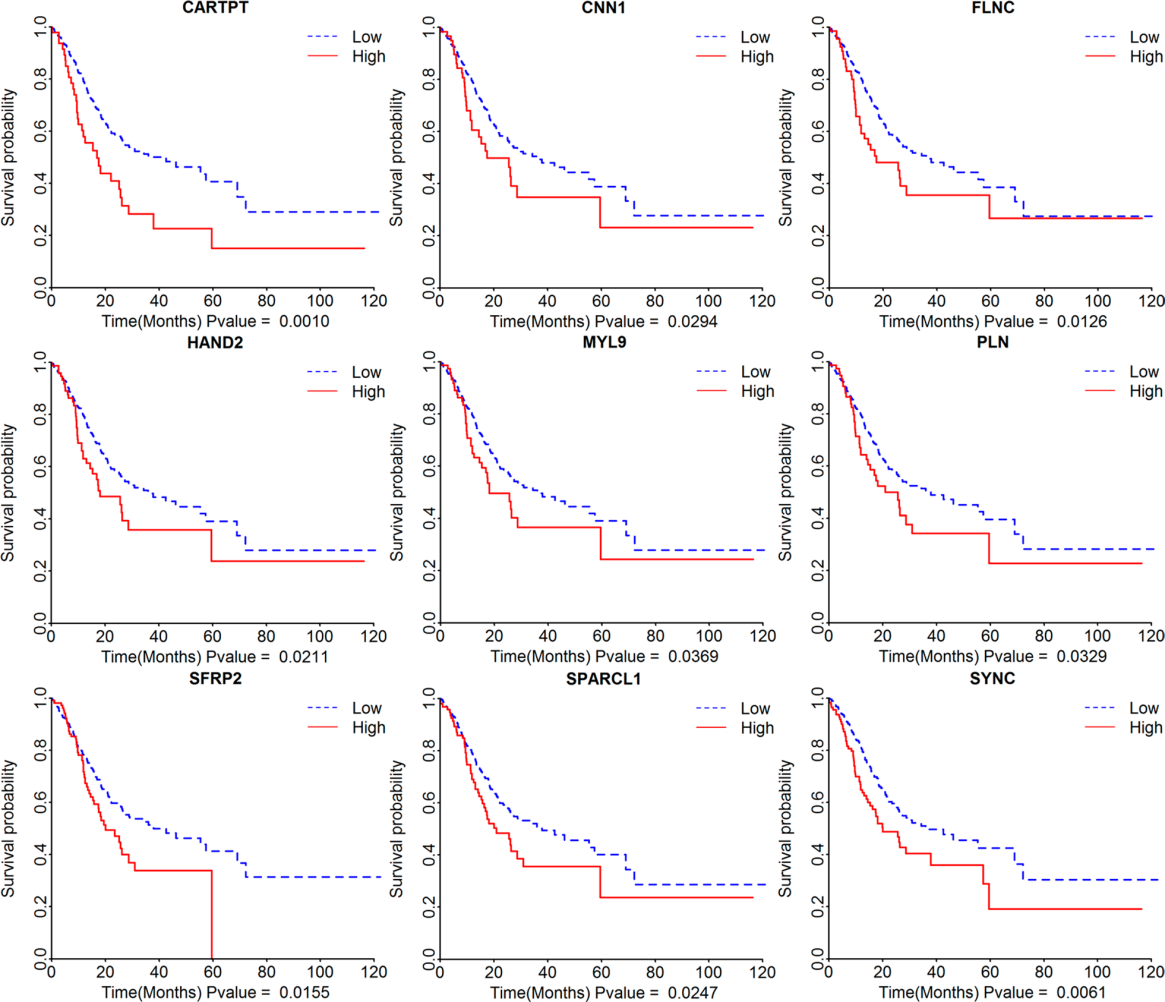
Survival analysis of differential genes. The Kaplan–Meier survival curves show the correlations between the expression levels of differential genes and the overall survival times (p < 0.05 in Log-rank test)

To analyse the potential role of differential genes in the overall survival of STAD patients, we downloaded STAD clinical data and gene expression data from the cBioportal website. Among all the differential genes, the high expression of 82 genes was associated with poor overall survival, and low expression of 9 genes (Fig. 3, p < 0.05) showed good overall survival (Additional file 2: Table S2).

### PPI network analysis of genes with prognostic values

**Fig. 4:**
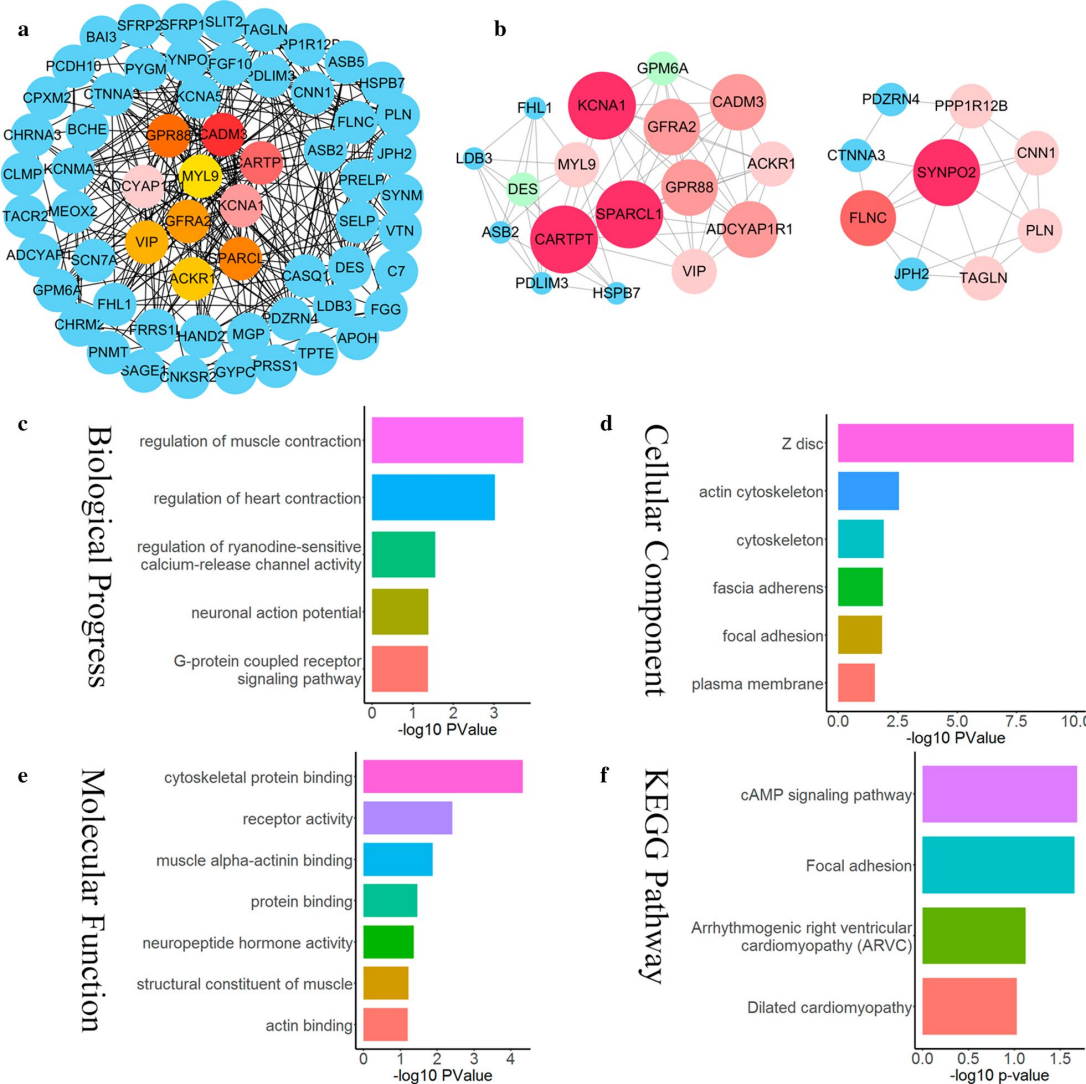
PPI network analysis of differential genes. **a** The network was constructed using the CytoHubba plug-in in Cytoscape, and the core gene scores were calculated using the Maximal Clique Centrality (MCC) method. The deeper the colour of the node, the higher the score. **b** Module analysis of PPI network. **c** Biological process, **d** cellular component, **e** molecular function, **f** KEGG pathways

To analyse the interrelationship between genes with prognostic value, we used the STRING network tool to construct a PPI network of genes with prognostic values. Core genetic analysis of the PPI network was performed using the CytoHubba plugin in Cytoscape software. The top 10 core genes extracted from the PPI network are: CADM3, CARTPT, KCNA1, ADCYAP1R1, GPR88, SPARCL1, GFRA2, VIP, ACKR1, and MYL9, the core score is up to 47,964 (Additional file 3: Table S3), the module contains 62 nodes and 317 edges (Fig. 4a). At the same time, we used Cytoscape’s MCODE plug-into perform a modular analysis of the differential genes. CARPTT and SYNPO2 modules were identified through module analysis. The CARTPT module contains 17 points and 66 edges, while the SYNPO2 module contains 9 points and 21 edges. In the CARTPT module, CARTPT, KCNA1, and SPARCL1 have higher degree values, while in the SYNPO2 module, SYNPO2 and FLNC have higher degree values (Fig. 4b).

We performed functional enrichment analysis on the genes mined by the PPI network module. There are four terms of the biological process: regulation of heart/muscle contraction, regulation of ryanodine-sensitive calcium-release channel activity, neuronal action potential, G-protein coupled receptor signalling pathway (Fig. 4c). There are six terms of the cellular component: Z disc, actin cytoskeleton, cytoskeleton, fascia adherents, focal adhesion, and plasma membrane (Fig. 4d). The molecular function contains seven terms: cytoskeletal protein binding, receptor activity, muscle alpha-actinin binding, protein binding, neuropeptide hormone activity, structural constituent of muscle, and actin binding (Fig. 4e). There are four terms of the KEGG pathway: cAMP signalling pathway, focal adhesion, arrhythmogenic right ventricular cardiomyopathy (ARVC), and dilated cardiomyopathy (Fig. 4f).

### Using the GEO database to verify genes with prognostic values

**Fig. 5:**
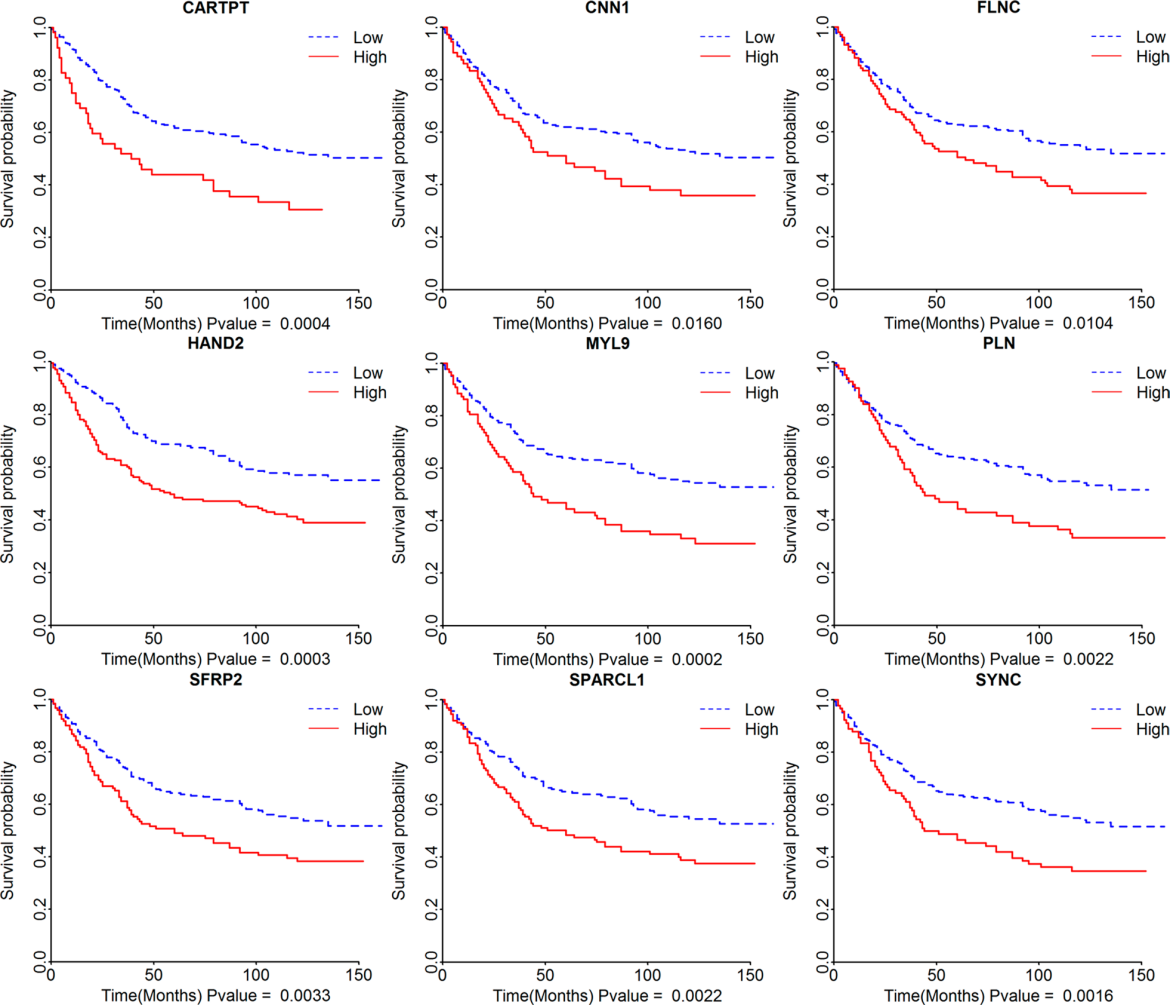
Validation of prognostic genes using data from the GEO database. Similar to TCGA results, in the GEO data analysis, high gene expression is associated with poor overall survival (p < 0.05 in Log-rank test)

To reveal whether the differential genes from TCGA database have an equal prognostic value in other STAD cases, we downloaded the GSE84433 expression dataset and clinical data from the GEO database, which contained 357 samples. A total of 31 genes (Additional file 4: Table S4) with high expression and poor prognosis were verified (Fig. 5), and 16 genes have not been reported to be associated with a poor prognosis for STAD (Table 1). These genes may be potential genes for poor prognosis of STAD and may provide some reference value for the treatment of STAD in the future.

**Table 1:** Genes that influence the overall survival of STAD in both TCGA and GEO

Categories	Gene symbols
Membrane	ACKR1, ADGRB3, *CHRNA3* [23], *CYP1B1* [24], *FLNC* [25], PLIN4, PLN, RNF150, *SELP* [26], TACR2
Extracellular region	*ANGPTL1* [27], BCHE, C7, *CARTPT* [28], *CPXM2* [29], PRG4, VIP
Extracellular matrix	*OMD* [30], *MGP* [31], KERA, *SFRP2* [32], *SPARCL1* [33]
Cytoplasm	*PDLIM3* [34], *MYL9* [35], *FHL1* [36], DES, SYNC, CPED1
Protein binding, DNA binding	HAND2, *CNN1* [37], BNC2

Italic genes have been reported to be associated with the overall survival prognosis of STAD

### Prognostic gene validation using clinical tissue samples

**Fig. 6:**
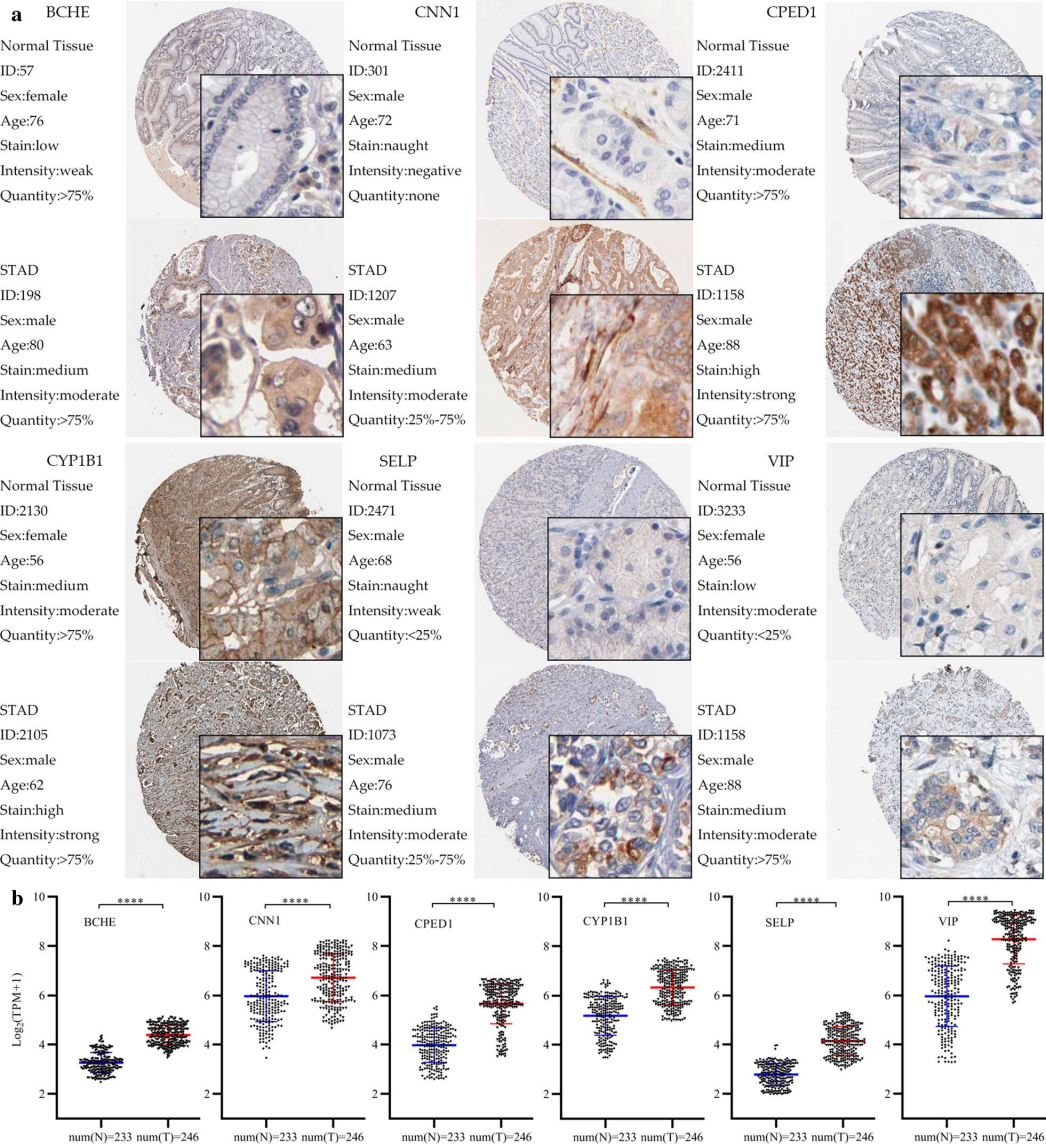
IHC analysis and RNA expression analysis of genes with prognostic values. **a** Differentially expressed proteins of genes with prognostic values in STAD and normal tissues in The Human Protein Atlas database. **b** RNA expression of genes with prognostic values between STAD and normal tissues in The Human Protein Atlas database. Significance tested by t-test (****p < 0.0001; num(N), Normal sample size; num(T), Tumor sample size)

To further confirm the reliability of the obtained genes with prognostic values, we used IHC to detect the protein expression of 31 genes in normal tissues and tumour tissues. The results showed that compared with normal tissues, sixteen proteins (ANGPTL1, BCHE, BNC2, CHRNA3, CNN1, CPED1, CYP1B1, FHL1, MYL9, PDLIM3, PRG4, RNF150, SELP, SPARCL1, SYNC, VIP) were significantly overexpressed in tumour tissues (Fig. 6, p < 0.05). The IHC map of the other 10 genes is shown in Additional file 5: Figure S1.

## Discussion

In this paper, the ESTIMATE algorithm [17] was used to calculate the stromal/immune scores of RNA-seq data of STAD in TCGA database. Subsequently, the STAD samples were divided into high and low score groups according to the stromal/immune scores. Through differential and downstream analysis, 82 high expression genes related to poor prognosis and 9 low expression genes related to good prognosis were obtained. Then, 31 of the above genes were verified by the data in the GEO database, and 15 genes have been reported to be linked to the overall survival in STAD.

First, we obtained 245 up-regulated genes and 103 down-regulated genes by analysing the differences between high- and low-score group samples. Following functional enrichment analysis of the up- and down-regulated genes, it was found that many genes are associated with the TME, which is consistent with previous studies reporting the important roles of immune cells and stromal cells in the TME [38–41]. A total of 10 core genes were extracted from the PPI network, and a module analysis was performed. The functional enrichment analysis of these genes revealed them to be mainly related to the immune and inflammatory response.

Next, we performed survival analysis on the differential genes. High expression of 91 genes was linked to poor overall survival. The above genes were validated using data from stomach adenocarcinoma patients in the GEO database, and 31 genes with poor prognosis were obtained, which could be used as potential biomarkers for future treatment of STAD. Combined with the PPI network, we focused on the two genes CARTPT and SPARCL1, which have the highest degree value. The full name of CARTPT is CART prepropeptide, and this gene encodes a preproprotein that is proteolytically processed to generate multiple biologically active peptides. These peptides play a role in appetite, energy balance, maintenance of body weight, reward and addiction, and the stress response. Expression of a similar gene transcript in rodents is up-regulated following administration of cocaine and amphetamine. Mutations in this gene are associated with susceptibility to obesity in humans [42, 43]. In the gastrointestinal mucosa, CART expression was mainly identified in gastrin-producing G cells, but the physiological function of CART in gastrointestinal endocrine cells has not been elucidated [44, 45]. SPARC1 is a member of the SPARC family, a member of the extracellular matrix glycoprotein, and is involved in many physiological functions [33]. It has been shown to be down-regulated in a variety of cancers and can be used as a negative regulator of cell growth and proliferation. With the promotion of invasion and tumour formation, changes in SPARC expression are associated with disease progression and poor prognosis [46, 47].

In addition, we performed IHC analysis on prognostic genes, and further confirmed gene expression patterns at the protein level based on the human protein map. The results showed that these 16 genes were highly expressed in STAD, suggesting that most of these genes may play a carcinogenic role in STAD.

There have been many experimental studies on the correlation between gene expression and survival of STAD patients, but the size of the subjects is generally small, lacking a more comprehensive analysis of STAD and its microenvironment [48, 49]. With the rapid development of sequencing technology, more and more tumour databases have been developed, such as TCGA, GEO, and can be used free of charge [50, 51]. They facilitate the large-scale and comprehensive analysis of data. The TME plays an important role in the development of tumours and affects their occurrence, growth, and metastasis [52–55]. Based on immunological and stromal cell analysis of STAD samples, we obtained TME-related genes with prognostic value, providing potential value for future treatment of STAD.

## Conclusions

In summary, the ESTIMATE algorithm was used to obtain immune/stromal scores for SA samples in TCGA database, which in turn yielded some prognostic genes associated with the TME. These genes were validated using data from the GEO database and may help outline the prognosis of STAD patients. Among them, the unreported genes could become potential biomarkers for STAD. In addition, research on the prognostic role of the overall gene set may provide significant information on their clinical applicability. Finally, further research on these genes may provide new insights into the TME in STAD with the potential of yielding better clinical outcomes.

## Supplementary information

**Additional file 1: Table S1.** Up and down-regulate differential genes.

**Additional file 2: Table S2.** Genes related to survival in STAD.

**Additional file 3: Table S3.** Top 10 core genes from PPI network.

**Additional file 4: Table S4.** Genes related to survival that have been verified in gastric cancer samples from the GEO database.

**Additional file 5: Figure S1.** IHC analysis of 10 other genes.

## Data Availability

The data that support the findings of this study are available in TCGA at https://portal.gdc.cancer.gov, reference number TCGA-STAD, and GEO dataset [GSE84433].

## Abbreviations

STAD: Stomach adenocarcinoma
TCGA: The Cancer Genome Atlas
TME: Tumour microenvironment
ECM: Extracellular matrix
GEO: Gene Expression Omnibus
DAVID: Database for Annotation, Visualization and Integrated Discovery
DEGs: Differentially expressed genes
BP: Biological processes
CC: Cell components
MF: Molecular functions
GO: Gene Ontology
KEGG: Kyoto Encyclopedia of Genes and Genomes
PPI: Protein–protein interaction
STRING: Search Tool for the Retrieval of Interacting Genes
MCODE: Molecular Complex Detection

## References

[1] Bray F, Ferlay J, Soerjomataram I, Siegel RL, Torre LA, Jemal A (2018). Global cancer statistics 2018: GLOBOCAN estimates of incidence and mortality worldwide for 36 cancers in 185 countries. CA Cancer J Clin.

[2] Siegel RL, Miller KD, Jemal A (2019). Cancer statistics, 2019. CA Cancer J Clin.

[3] Van Cutsem E, Sagaert X, Topal B, Haustermans K, Prenen H (2016). Gastric cancer. Lancet.

[4] Zhu YH, Jeong S, Wu M, Jin ZY, Zhou JY, Han RQ (2019). Dietary intake of fatty acids, total cholesterol, and stomach cancer in a Chinese population. Nutrients..

[5] Parkin DM, Bray F, Ferlay J, Pisani P (2005). Global cancer statistics, 2002. CA Cancer J Clin.

[6] Allemani C, Matsuda T, Di Carlo V, Harewood R, Matz M, Nikšić M (2018). Global surveillance of trends in cancer survival 2000–14 (CONCORD-3): analysis of individual records for 37 513 025 patients diagnosed with one of 18 cancers from 322 population-based registries in 71 countries. Lancet.

[7] Jia D, Li S, Li D, Xue H, Yang D, Liu Y (2018). Mining TCGA database for genes of prognostic value in glioblastoma microenvironment. Aging (Albany NY)..

[8] Kankeu Fonkoua L, Yee NS (2018). Molecular characterization of gastric carcinoma: therapeutic implications for biomarkers and targets. Biomedicines..

[9] Zeng D, Li M, Zhou R, Zhang J, Sun H, Shi M (2019). Tumor microenvironment characterization in gastric cancer identifies prognostic and immunotherapeutically relevant gene signatures. Cancer Immunol Res.

[10] Binnewies M, Roberts EW, Kersten K, Chan V, Fearon DF, Merad M (2018). Understanding the tumor immune microenvironment (TIME) for effective therapy. Nat Med.

[11] Hanahan D, Weinberg RA (2000). The hallmarks of cancer. Cell.

[12] Hanahan D, Coussens LM (2012). Accessories to the crime: functions of cells recruited to the tumor microenvironment. Cancer Cell.

[13] Jiang X, Wang J, Deng X, Xiong F, Ge J, Xiang B (2019). Role of the tumor microenvironment in PD-L1/PD-1-mediated tumor immune escape. Mol Cancer..

[14] Ren Q, Zhu P, Zhang H, Ye T, Liu D, Gong Z (2020). Identification and validation of stromal-tumor microenvironment-based subtypes tightly associated with PD-1/PD-L1 immunotherapy and outcomes in patients with gastric cancer. Cancer Cell Int.

[15] Yin Z, Lan H, Tan G, Lu M, Vasilakos AV, Liu W (2017). Computing platforms for big biological data analytics: perspectives and challenges. Comput Struct Biotechnol J..

[16] He KY, Ge D, He MM (2017). Big data analytics for genomic medicine. Int J Mol Sci.

[17] Yoshihara K, Shahmoradgoli M, Martínez E, Vegesna R, Kim H, Torres-Garcia W (2013). Inferring tumour purity and stromal and immune cell admixture from expression data. Nat Commun.

[18] Xu WH, Xu Y, Wang J, Wan FN, Wang HK, Cao DL (2019). Prognostic value and immune infiltration of novel signatures in clear cell renal cell carcinoma microenvironment. Aging (Albany NY)..

[19] Alonso MH, Ausso S, Lopez-Doriga A, Cordero D, Guino E, Sole X (2017). Comprehensive analysis of copy number aberrations in microsatellite stable colon cancer in view of stromal component. Br J Cancer.

[20] da Huang W, Sherman BT, Lempicki RA (2009). Systematic and integrative analysis of large gene lists using DAVID bioinformatics resources. Nat Protoc.

[21] Szklarczyk D, Franceschini A, Wyder S, Forslund K, Heller D, Huerta-Cepas J (2015). STRING v10: protein-protein interaction networks, integrated over the tree of life. Nucleic Acids Res..

[22] Shannon P, Markiel A, Ozier O, Baliga NS, Wang JT, Ramage D (2003). Cytoscape: a software environment for integrated models of biomolecular interaction networks. Genome Res.

[23] Wu H, Wang Y, Wang S, Jiang M, Wang C, Fu W (2013). Is susceptibility locus for lung cancer in the 15q25 nicotinic acetylcholine receptor gene cluster CHRNA5-A3-B4 associated with risk of gastric cancer?. Med Oncol.

[24] Hiraki M, Kitajima Y, Koga Y, Tanaka T, Nakamura J, Hashiguchi K (2011). Aberrant gene methylation is a biomarker for the detection of cancer cells in peritoneal wash samples from advanced gastric cancer patients. Ann Surg Oncol.

[25] Shi J, Zhang G, Yao D, Liu W, Wang N, Ji M (2012). Prognostic significance of aberrant gene methylation in gastric cancer. Am J Cancer Res..

[26] Lan X, Xing J, Gao H, Li S, Quan L, Jiang Y (2017). Decreased expression of selenoproteins as a poor prognosticator of gastric cancer in humans. Biol Trace Elem Res.

[27] Jiang H, Gu J, Du J, Qi X, Qian C, Fei BJMMR (2020). A 21-gene support vector machine classifier and a 10-gene risk score system constructed for patients with gastric cancer. Mol Med Rep.

[28] Zhang C, Liang Y, Ma MH, Wu KZ, Dai DQ (2019). KRT15, INHBA, MATN3, and AGT are aberrantly methylated and differentially expressed in gastric cancer and associated with prognosis. Pathol Res Pract.

[29] Niu G, Yang Y, Ren J, Song T, Hu Z, Chen L (2019). Overexpression of CPXM2 predicts an unfavorable prognosis and promotes the proliferation and migration of gastric cancer. Oncol Rep.

[30] Wang Z, Chen G, Wang Q, Lu W, Xu M (2017). Identification and validation of a prognostic 9-genes expression signature for gastric cancer. Oncotarget..

[31] Zhang J, Liu X, Yu G, Liu L, Wang J, Chen X (2018). UBE2C is a potential biomarker of intestinal-type gastric cancer with chromosomal instability. Front Pharmacol..

[32] Wang H, Duan XL, Qi XL, Meng L, Xu YS, Wu T (2017). Concurrent hypermethylation of SFRP2 and DKK2 activates the Wnt/beta-catenin pathway and is associated with poor prognosis in patients with gastric cancer. Mol Cells.

[33] Li P, Qian J, Yu G, Chen Y, Liu K, Li J (2012). Down-regulated SPARCL1 is associated with clinical significance in human gastric cancer. J Surg Oncol.

[34] Deng X, Xiao Q, Liu F, Zheng C (2018). A gene expression-based risk model reveals prognosis of gastric cancer. PeerJ..

[35] Sun C, Yuan Q, Wu D, Meng X, Wang B (2017). Identification of core genes and outcome in gastric cancer using bioinformatics analysis. Oncotarget..

[36] Xu Y, Liu Z, Guo K (2012). Expression of FHL1 in gastric cancer tissue and its correlation with the invasion and metastasis of gastric cancer. Mol Cell Biochem.

[37] Bai Z, Ye Y, Liang B, Xu F, Zhang H, Zhang Y (2011). Proteomics-based identification of a group of apoptosis-related proteins and biomarkers in gastric cancer. Int J Oncol.

[38] Kim JW, Nam KH, Ahn SH, Park DJ, Kim HH, Kim SH (2016). Prognostic implications of immunosuppressive protein expression in tumors as well as immune cell infiltration within the tumor microenvironment in gastric cancer. Gastric Cancer.

[39] Bussard KM, Mutkus L, Stumpf K, Gomez-Manzano C, Marini FC (2016). Tumor-associated stromal cells as key contributors to the tumor microenvironment. Breast Cancer Res.

[40] Choi Y, Kim JW, Nam KH, Han SH, Kim JW, Ahn SH (2017). Systemic inflammation is associated with the density of immune cells in the tumor microenvironment of gastric cancer. Gastric Cancer.

[41] Yu PC, Long D, Liao CC, Zhang S (2018). Association between density of tumor-infiltrating lymphocytes and prognoses of patients with gastric cancer. Medicine (Baltimore)..

[42] Lau J, Herzog H (2014). CART in the regulation of appetite and energy homeostasis. Front Neurosci..

[43] Cutcutache I, Wu AY, Suzuki Y, McPherson JR, Lei Z, Deng N (2016). Abundant copy-number loss of CYCLOPS and STOP genes in gastric adenocarcinoma. Gastric Cancer.

[44] Ekblad E, Kuhar M, Wierup N, Sundler F (2003). Cocaine- and amphetamine-regulated transcript: distribution and function in rat gastrointestinal tract. Neurogastroenterol Motil.

[45] Volkoff H, Peter RE (2001). Characterization of two forms of cocaine- and amphetamine-regulated transcript (CART) peptide precursors in goldfish: molecular cloning and distribution, modulation of expression by nutritional status, and interactions with leptin. Endocrinology.

[46] Gagliardi F, Narayanan A, Mortini P (2017). SPARCL1 a novel player in cancer biology. Crit Rev Oncol Hematol.

[47] Li T, Liu X, Yang A, Fu W, Yin F, Zeng X (2017). Associations of tumor suppressor SPARCL1 with cancer progression and prognosis. Oncol Lett..

[48] Wang Q, Hu B, Hu X, Kim H, Squatrito M, Scarpace L (2018). Tumor evolution of glioma-intrinsic gene expression subtypes associates with immunological changes in the microenvironment. Cancer Cell.

[49] Shigemori T, Toiyama Y, Okugawa Y, Yamamoto A, Yin C, Narumi A (2019). Soluble PD-L1 expression in circulation as a predictive marker for recurrence and prognosis in gastric cancer: direct comparison of the clinical burden between tissue and serum PD-L1 expression. Ann Surg Oncol.

[50] Li J, Lu Y, Akbani R, Ju Z, Roebuck PL, Liu W (2013). TCPA: a resource for cancer functional proteomics data. Nat Methods.

[51] Chen J, Kong Y, Weng S, Dong C, Zhu L, Yang Z (2017). Outcomes of surgery for gastric cancer with distant metastases: a retrospective study from the SEER database. Oncotarget..

[52] Thakkar S, Sharma D, Kalia K, Tekade RK (2019). Tumor microenvironment targeted nanotherapeutics for cancer therapy and diagnosis: a review. Acta Biomater.

[53] Taube JM, Galon J, Sholl LM, Rodig SJ, Cottrell TR, Giraldo NA (2018). Implications of the tumor immune microenvironment for staging and therapeutics. Mod Pathol.

[54] Petitprez F, Vano YA, Becht E, Giraldo NA, de Reynies A, Sautes-Fridman C (2018). Transcriptomic analysis of the tumor microenvironment to guide prognosis and immunotherapies. Cancer Immunol Immunother.

[55] Wiggins JM, Opoku-Acheampong AB, Baumfalk DR, Siemann DW, Behnke BJ (2018). Exercise and the tumor microenvironment: potential therapeutic implications. Exerc Sport Sci Rev.

