# Supplementary figures and images for: Exploring TCGA database for identification of potential prognostic genes in stomach adenocarcinoma
Source: Cancer Cell Int. 2020 Jun 23;20:264. doi: 10.1186/s12935-020-01351-3 (PMC7310509; doi:10.1186/s12935-020-01351-3)

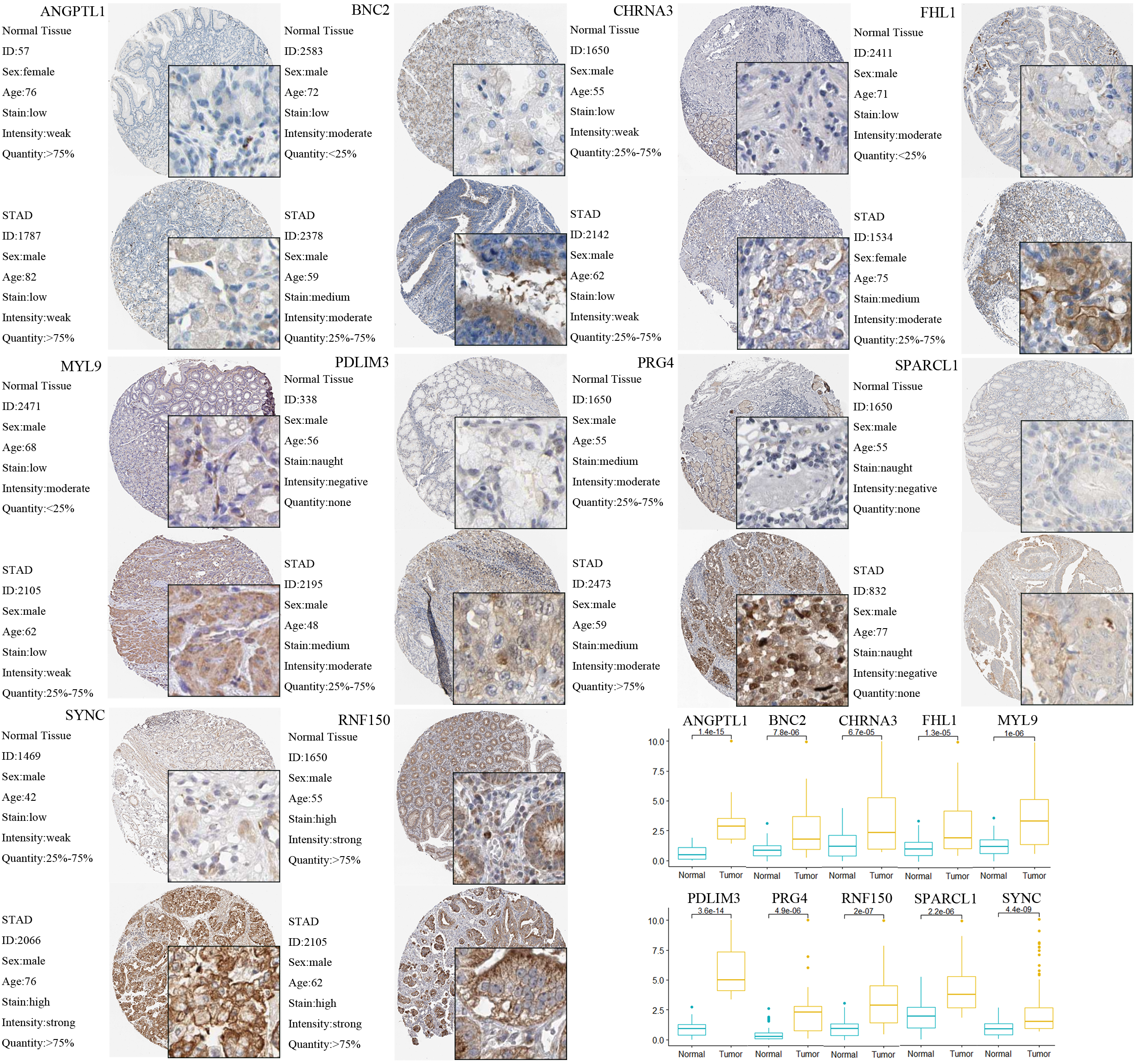

Supplement: Supplementary file 5 — Additional file 5: Figure S1. IHC analysis of 10 other genes. [file 12935_2020_1351_MOESM5_ESM.tif]
